# Everybody's Page

**Published:** 1888-12-01

**Authors:** 


### Everybody's Page.

Dr. De Chaumont was of opinion that 857 cubic feet of air-space was the minimum a human being could do with, while Dr. Parkes thought 3,000 cubic feet per head per hour was necessary for healthy sleep.

In New York, several patients suffering from poisoning by gas have been bled copiously, with good effect. One woman, with whom all other remedies seemed hopeless, recovered rapidly after being robbed of twenty ounces of blood.

The doctors of mediæval times seem to have understood the fact that leprosy was so far like scurvy that good nourishing diet, with a fair amount of fresh vegetables, was one of the first essentials to its successful treatment. The diet-sheets of the old English lazar houses contain a list of articles absolutely luxurious for the times, and not by any means despicable according to modern ideas. Considering how nauseous mediæval medicines were, it is good to find that the food given to the sick in those days was good and plentiful.

To our already formidable list of poisons may be added, as a source of occasional danger, the fragrant nutmeg. It is not likely that adults will ever die of eating nutmegs, but the inquisitive fingers of children find their way to the spice-box, and thence convey many things to their curious palates. At least one fatal case has occurred, where a boy of eight, having eaten two nutmegs, fell into a comatose condition, and died within twelve hours. The symptoms were similar to those of opium poisoning.

The Empress Catherine II. of Russia deserves the credit of having established hospitals for cases of infectious disease. She issued an Imperial ukase in 1763, commanding the erection of these in all cities and large towns. It was she who established the Foundling Hospital in Moscow in 1764, as well as a Home for the Aged and a Hospital for Chronic Diseases in St. Petersburg. She also instituted an Asylum for the Insane in 1779, and was the first Russian monarch to set on foot a Public Medical Service.

The "fiery serpents" which tortured the children of Israel in the desert are generally supposed to be identical with the Aracunculus or Guinea-worm, which is one of the plagues of modern India. This animal finds its favourite habitat in the skin of the lower extremities of human beings, and while it is so thin that it has been called the "hair worm," it attains an average length of twenty-four inches. The presence of the parasite sets up inflammations and suppurations of a nature so painful as sufficiently to justify the fiery soubriquet, and it is obstinately difficult to extract.

Most people know the epigrammatic definition of the qualities of most extracts of meat which was given by an American analyst. He said that, in his experience, they were almost as good as brown sugar for colouring gravies, but not quite. It is true that most of the extracts, bouillons, and liquid beefs offered to the public contain very little beef indeed. Very few of them are at all calculated to offend the susceptibilities of that enthusiastic vegetarian who said he would rather lay his head on the block than become a carnivorous animal. But the Liebig Company's extract is not one of these. The Company's analysts state, indeed, that "it contains from three to six times as much of the real essence of meat as its rivals and imitators, and moreover contains no added salt, whereas most of them contain from 50 to 75 per cent. of that condiment—a very serious consideration, when one remembers that salt meat has very much less nutritive power than fresh." In this connection we must also affirm that impartial trials and long use have abundantly justified the great popularity of Brand's excellent essence of beef, and of the useful concentrated beef tea prepared by this firm.

Sleep is the brain's method of repair. The brain-cells lose more and more of their substance during waking hours, and if sleep does not come, mere loss is supplemented by absolute disintegration. When this takes place, the cells not only fail to perform their part in the work of the mind, they lose their capability for repair, and cause permanent disorder or enfeeblement of the intellect. Prolonged want of rest is apt, moreover, to lead to a habit of sleeplessness, which is highly dangerous to mental health. In the old days, when criminals were kept from sleeping between their condemnation and their execution, they almost invariably became mad.

A physician who has been practising on the West Coast of Africa gives the following sensible advice to those who think of following in his footsteps: "Take a supply of remedies for your personal use, and use them on the slightest provocation. Dress warmly; don't think because you are in a torrid climate you can go half naked. Bathe night and morning, and eat fresh food; tinned meats cause more deaths in the tropics than anything else. Don't be ashamed to come home if you get ill. I would rather be ridiculed as a live man than mourned for as a corpse." This gentleman would not despise the prudent Hibernian who recently said he would rather be a coward for five minutes than a corpse for the rest of his life.

There can be little doubt that the air and water in the neighbourhood of cemeteries becomes in many instances poisonous in the extreme. Persons breathing this air or drinking this water have their vital power lowered, even if they escape actual disease, and are always subject to headaches and ulcerated sore throats. It is said that the putrid emanations from the Parisian cemeteries of Père la Chaise, Montmartre, and Mont Parnasso have caused serious disease of throat and lungs, which destroy large numbers of persons yearly. One throat disease, which often kills those affected by it in a few hours, has been traced to the absorption of vitiated air into the windpipe, and is most common near burying grounds.

Who says that women are hysterical, timorous, and prone to imagine themselves ill when they are in perfect health? A good many men do say all this, but among them will not be numbered Dr. Durand, of New Orleans. An experiment he recently made has proved to him which is, medically speaking, the saner sex. Being anxious to test the power of the imagination on the health, he gave to each of twenty patients a dose of sweetened water. A quarter of an hour afterwards he announced to them that he had administered a powerful emetic. Fifteen of the patients got excited and nervous, and immediately displayed the usual symptoms that follow the administration of such a drug. Five were unaffected by the announcement. And the excitable fifteen were men, and the placid five were women.

It is strange that some nurses and parents should look almost with satisfaction on the eruptions that sometimes disfigure the skin of children. "It's better out than in," they say; "it" being a supposed impurity of the blood that thus betrays itself on the epidermis. They would be much surprised to hear that in nine cases out of ten the cause of "it" is insufficient or improper food. Food that is ample in quantity may be starvation diet all the same, if it be not sufficiently varied to contain all the substances required by the body. In many cases it is fatty matters that are wanted, and the application of cod-liver oil to the irritated skin, when combined with more and richer milk and fatter meat in the daily diet, will effect a transformation, and make "it" disappear from both the surface and the tissues of the body.

